# Effect of Small-Scale Turbulence on the Physiology and Morphology of Two Bloom-Forming Cyanobacteria

**DOI:** 10.1371/journal.pone.0168925

**Published:** 2016-12-30

**Authors:** Yan Xiao, Zhe Li, Chao Li, Zhen Zhang, Jinsong Guo

**Affiliations:** 1 CAS Key Laboratory on Reservoir Water Environment, Chongqing Institute of Green and Intelligent Technology, Chinese Academy of Sciences, Chongqing, China; 2 State Key Laboratory of Bioreactor Engineering, College of Biotechnology, East China University of Science and Technology, Shanghai, China; National Taiwan Ocean University, TAIWAN

## Abstract

The main goal of the present work is to test the hypothesis that small-scale turbulence affected physiological activities and the morphology of cyanobacteria in high turbulence environments. Using quantified turbulence in a stirring device, we conducted one set of experiments on cultures of two strains of cyanobacteria with different phenotypes; i.e., unicellular *Microcystis flos-aquae* and colonial *Anabaena flos-aquae*. The effect of small-scale turbulence examined varied from 0 to 8.01×10^−2^ m^2^s^-3^, covering the range of turbulence intensities experienced by cyanobacteria in the field. The results of photosynthesis activity and the cellular chlorophyll *a* in both strains did not change significantly among the turbulence levels, indicating that the potential indirect effects of a light regime under the gradient of turbulent mixing could be ignored. However, the experiments demonstrated that small-scale turbulence significantly modulated algal nutrient uptake and growth in comparison to the stagnant control. Cellular N and C of the two stains showed approximately the same responses, resulting in a similar pattern of C/N ratios. Moreover, the change in the phosphate uptake rate was similar to that of growth in two strains, which implied that growth characteristic responses to turbulence may be dependent on the P strategy, which was correlated with accumulation of polyphosphate. Additionally, our results also showed the filament length of *A*. *flos-aquae* decreased in response to high turbulence, which could favor enhancement of the nutrient uptake. These findings suggested that both *M*. *flos-aquae* and *A*. *flos-aquae* adjust their growth rates in response to turbulence levels in the ways of asynchronous cellular stoichiometry of C, N, and P, especially the phosphorus strategy, to improve the nutrient application efficiency. The fact that adaptation strategies of cyanobacteria diversely to turbulence depending on their physiological conditions presents a good example to understand the direct cause—effect relationship between hydrodynamic forces and algae.

## Introduction

Accelerated eutrophication of freshwater bodies often leads to the excessive proliferation of phytoplankton, of which cyanobacteria tend to predominate. Rapid growth of cyanobacteria often leads to blooms that can have serious adverse effects on aquatic communities in lakes throughout the world [1]. Cyanobacteria bloom occurrence is affected by a complex set of physical, chemical, biological, hydrological, and meteorological conditions, so it is difficult to isolate specific causative environmental factors [2]. Previous investigations suggested that interactions among nutrient inputs, turbulent mixing, underwater light availability, and grazing pressure can strongly influence the components of phytoplankton species in the water column [3, 4].

Turbulence mixing has been widely accepted as a source of external energy to the planktonic environment, such as regulating light accessibility in the water column [5], bringing eutrophic water into contact with algal cells [6, 7], and affecting encounter possibilities between phytoplankton and predators [8]. In particular, small-scale turbulence could play an important role in determining which phytoplanktonic taxon dominates over other competitors in the ever-changing environment [9]. Phytoplankton in the water column are constantly exposed to small-scale turbulence, which may have a differential effect on the physiology of algae [10]. The positive effect of small-scale turbulence on phytoplankton has been reported to occur through changing diffusive sublayers and regulating nutrient fluxes of cells [11–13], while the negative impacts include mechanical damage, behavioral alteration and physiological impairment [10, 14–16]. However, to date, there is only a small body of literature on the direct effects of small-scale turbulence on cyanobacteria in the freshwater rivers and lakes.

Cyanobacteria have constantly been subjected to a complex field environment imposed by these small-scale turbulent conditions. Survival of different cyanobacteria species in the mixed water column depends on the combined effects of varying environmental factors [17]. Nutrients and light are crucial, but the turbulence and its intensity also affect growth strategies of cyanobacteria species in their competition for these basic commodities [18, 19]. Because of a variety of adaptive strategies, such as buoyancy [20], N_2_-fixation [21], light tolerance [22], and efficient utilization of phosphorus and inorganic carbon [23, 24], cyanobacteria can dominate over other competitors throughout the life cycle. Under certain environmental stressors, cyanobacteria adapt habitat energy and nutrients through the adjustment of their metabolism while keeping cellular carbon, nitrogen and phosphorus in an acceptable range [25, 26]. Nevertheless, this plasticity of adaptation regarding nutrients has not been well reported when cyanobacteria species encounter turbulence mixing.

Many studies have reported that heterogeneity in response to turbulence is often observed among phytoplankton taxa [27], and certain species may exhibit specific adaptive strategies to turbulence. For instance, genus-specific shear effects have been illustrated for cyanobacteria such that the non-heterocystous filamentous *Oscillatoria* appears to be more shear tolerant than the heterocystous *Anabaena* [28, 29]. Furthermore, the seasonal field studies of mixed phytoplankton communities and enclosure experiments also have shown that shear has either increased or decreased/arrested cyanobacterial abundance or activity [28–30]. Some researchers suggested these differences in sensitivity induced by turbulence depend on cell size. It has been reported that the increase in the nutrient diffusion rate into cells due to small-scale turbulence varies from negligible to significant as cell size increases [13].

Hard evidence of a direct cause—effect relationships between turbulence and algae is not easy to find [8, 31]. In the present study, we investigated the effect of small-scale turbulence on the physiology and morphology of two bloom-forming cyanobacteria with different phenotypes, i.e., unicellular *Microcystis flos-aquae* and colonial *Anabaena flos-aquae*. To our knowledge, few experiments have been performed employing quantified turbulence levels to evaluate the effect of small-scale turbulence on the physiology of cyanobacteria. Thus we developed a model to quantify turbulence exposure in the experiment and systematically investigate the change in photosynthesis and total cellular C, N and P of the two stains, and filament length of *A*. *flos-aquae* under different turbulent dissipation rates. The data from the study were analyzed to illustrate the mechanisms of cyanobacteria with different morphology adaptation strategies under small-scale turbulence mixing.

## Materials and Methods

### Strains and culture conditions

Unicellular *M*. *flos-aquae* FACHB1028 and colonial *A*. *flos-aquae* FACHB245 were obtained from the Freshwater Algae Culture Collection of the Institute of Hydrobiology (FACHB-Collection, Wuhan, China). Routine cultures of the two strains were maintained in BG11 medium with a phosphate concentration of 2 mg/L [32] under a cool-white fluorescent light intensity of 25 μmol m^-2^ s^-1^ with a 12:12 light:dark cycle at a temperature of 25±1°C, and they were manually shaken 3 times every day during incubation.

**Fig 1:**
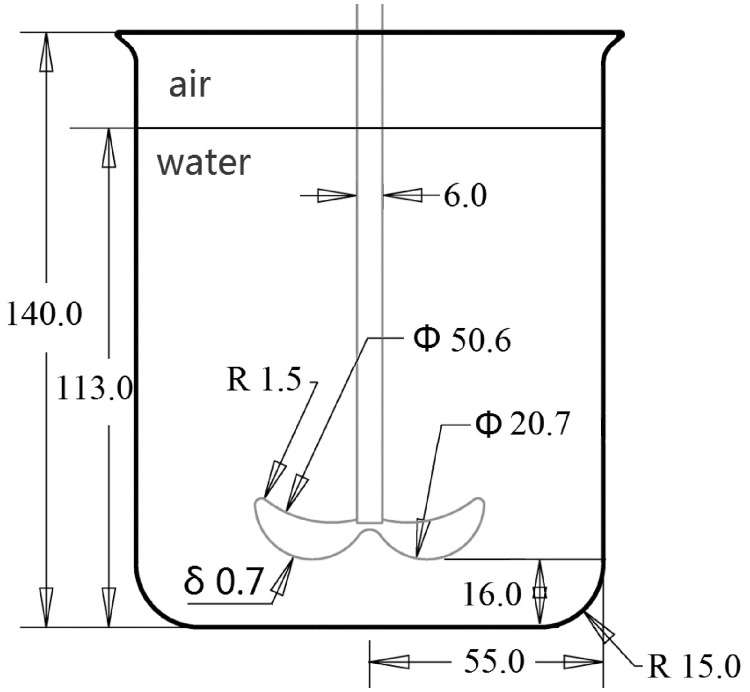
Geometric dimensions of the reactor (Unit: mm).

In the experiments, cells at their exponential growth phase were served with P free BG11 medium for a period of 10 days before the growing experiment. After that, they were inoculated into 1 L glass beakers with 2 mg/L phosphate concentration BG11 medium and exposed to different agitation intensities. A 1-L beaker was used as the reactor in the experiment. A self-designed impeller was located at the center of the beaker. Geometric dimensions are shown in Fig 1. The liquid height was 113 mm. Continuous stirring was performed at the start of the growing experiment at rotation speeds of 100, 200, 300, 400 and 500 rpm. The samples cultured in completely stagnant conditions were used as controls. The test was run in triplicate for two weeks under the conditions described above, and they were sampled every 2 days for the determination of varying parameters, as described below.

### Modeling and calculation of turbulent dissipation rates

A two-phase flow Computational Fluid Dynamics (CFD) model was used to simulate hydrodynamics in the reactor under different rotation speed [33]. The whole reactor was considered as the computational domain and was discretized using unstructured grids, where finer grids were performed in impeller and shaft regions (maximum size = 1 mm). About 1,000,000 total computational grids (maximum size = 2 mm) were created using the tetra mesh option of ANSYS ICEM CFD (ANSYS Inc.) in order to get the grid independent solution for the flow. The simulation of the process was considered as a steady-state, and numerically solved by the commercial software package CFX 12.0 (ANSYS Inc.). The convergence criteria used in all the simulations was 1×10^−4^, which was a factor by which the initial mass flow residual reduced as the simulation progressed. The simulations were carried out on the 6 nodes, 96 processor SUGON cluster. A commercial 2D particle image velocimetry (PIV) system (ILA, German), including a laser (Leamtech, Nd:YAG, 200Mj, 15Hz), a PIV camera (PCO2000, 2048×2048 pixels), a synchronizer and Vidpiv 4.7 software, was employed to validate the model. The setup and data processing were the same as described earlier [34]. Detail information of its validation results were reported in S1 File. The integrated results of turbulence mixing rate and Kolmogorov microscale in different levels of rotation speed were shown in Table 1.

**Table 1:** Turbulent dissipation rate and Kolmogorov microscale in different levels of rotation speed estimated by the CFD hydrodynamic model.

Turbulence					
**Rotation speed (rpm)**	100	200	300	400	500
**Turbulent dissipation rate (m**^**2**^**s**^**-3**^**)**	1.51×10^−3^	6.63×10^−3^	2.26×10^−2^	5.06×10^−2^	8.01×10^−2^
**Kolmogorov microscale (mm)**	0.186	0.126	0.092	0.074	0.065

### Determination of growth rates and chlorophyll *a* (*Chla*) of cyanobacteria

Two strains were batch cultured under different agitation intensities (0 rpm to 500 rpm) for two weeks and were sampled every 2 days for cell counting under a microscope (Olympus CX 43, Japan) using the appropriate magnification and a hemocytometer. Prior to cell counting, *A*. *flos-aquae* colonies were treated with ultrasonication for 30 s or 60 s at 20 kHz and 100 W (JY92-2D, SCIENTZ, China) to obtain single cells. The growth rate was measured during the exponential growth phase according to the exponential growth equation: μ = (lnBt_2_-lnBt_1_)/Δt [35], where μ is the specific growth rate, Bt_2_ and Bt_1_ represent the cell concentration at the end (t_2_) and beginning (t_1_) of the log phase, respectively, and Δt = t_2_-t_1_. For *Chla* measurements, the samples were centrifuged at 6,000 rpm for 15 min, and cell pellets were extracted with 80% acetone/20%water.

### Determination of photosynthetic activity of cyanobacteria

*In vivo* chlorophyll fluorescence was measured with a phytoplankton analyzer (PHYTO-PAM, Walz GmbH, Germany). The samples were dark-adapted for at least 15 min before measuring the fluorescence parameters (photosystem II activity, PSII). The electron transport rate of PSII (ETR) was calculated according to [36]. Relative ETR = (((*F*_*m*_*- F*_*t*_) */F*_*m*_)×0.84×0.5×PAR (m^-2^s^-1^)). The maximum effective quantum yield of PSII was calculated as *F*_*v*_*/F*_*m*_ = (*F*_*m*_*-F*_*0*_*)/F*_*m*_ [37]. *F*_*v*_ is the difference between *F*_*m*_ and *F*_*0*_, which are the maximum and minimum fluorescence values of the dark adapted stage of PSII.

### Analysis of the carbon, nitrogen and phosphorus contents of cyanobacteria

To measure the carbon, nitrogen and phosphorus contents of the two strains, culture suspensions were taken from each sample, filtered through GF/F filters (Whatman, UK) and dried at 65°C for 24 h. Each filter was split for analysis of C, N, and total cellular phosphorus (TCP). C and N concentrations were measured using a Vario El cube elemental analyzer (Elementar, Germany). The filtrates were used for measuring soluble reactive phosphorus (SRP). The filtered extracts were diluted with distilled water, and TCP was determined by digestion [38, 39] followed by colorimetric analysis [40]. The polyphosphate (polyP) was extracted using the hot water method according to [41].

### Uptake rate of phosphate

Phosphate uptake rates were calculated by the change in phosphate concentration in the culture media during 72 h batch cultures at the same culture conditions as mentioned above. Phosphate levels were determined using ascorbic acid-molybdenum blue spectrophotometric methods [42]. The uptake rate was calculated according to [43]: phosphate uptake rate = *(W*_*0*_*-W*_*t*_*) · V · N*_*0*_^*-1*^*· t*^*-1*^, where *W*_*0*_ is the initial concentration of phosphate, *W*_*t*_ is the concentration of the phosphate after *t* hours, *N*_*0*_ is the initial level of cell density and *V* is the volume of culture medium.

### Measurement of the filament size of *Anabaena*

*Anabaena* filaments were examined using an Olympus BX 53 light microscope with a digital camera (Olympus DP 71, Japan). The sizes of the filaments were measured in microphotographs using image analysis Olympus DP-Soft. One hundred filaments were chosen at random from each sample to capture the images, and then the surface area (S), width (d), and length (l) of individual filaments were obtained using Olympus DP-Soft, which was calibrated separately for each magnification.

### Statistical analysis

The data in this study are presented as the mean ± standard deviation (SD). Significant differences between controls and treated samples were determined with an ANOVA. All statistical analyses were carried out with Origin 8.0 (OriginLab, USA). Differences were considered to be significant at *P* < 0.05.

## Results

### Effect of turbulence on the growth rate of cyanobacteria

Specific growth rates of unicellular *M*. *flos-aquae* and colonial *A*. *flos-aquae* at different turbulent dissipation rates are shown in Table 2. The growth curve was plotted to roughly ascertain the period of the exponential phase of each strain, and thus the growth rates of individual strains were calculated in the exponential phase accordingly. For *M*. *flos-aquae*, the growth rate increased with the elevation of the turbulent dissipation rate. At a turbulent dissipation rate of 1.51×10^−3^ m^2^s^-3^, the growth rate of *M*. *flos-aquae* was approximately 3.4 times than that in the stagnation condition. When the turbulent dissipation rate was above 5.06×10^−2^ m^2^s^-3^, the growth rate of *M*. *flos-aquae* remained relatively unchanged (ANOVA, *P* > 0.05). On the other hand, the growth rate of *A*. *flos-aquae* increased dramatically (ANOVA, *P* < 0.05) at lower turbulent dissipation rates and reached the max at 2.26×10^−2^ m^2^s^-3^, while the growth rate decreased with increases in the turbulence intensity at higher levels. Overall, the growth rates of *A*. *flos-aquae* at all levels of turbulence intensity were lower than that of *M*. *flos-aquae*.

**Table 2:** The specific growth rates of *M*. *flos-aquae* and *A*. *flos-aquae* under different levels of turbulence.

Turbulent dissipation rate (m^2^s^-3^)						
Strain	0	1.51×10^−3^	6.63×10^−3^	2.26×10^−2^	5.06×10^−2^	8.01×10^−2^
***M*. *flos-aquae***	0.039±0.003	0.133±0.002	0.144±0.001	0.223±0.005	0.261±0.005	0.286±0.004
***A*. *flos-aquae***	0.009±0.001	0.014±0.002	0.027±0.001	0.043±0.002	0.040±0.003	0.029±0.003

The data are the mean ± SD (n = 3)

### Photosynthetic parameters and cellular *Chla* concentrations of cyanobacteria

**Fig 2:**
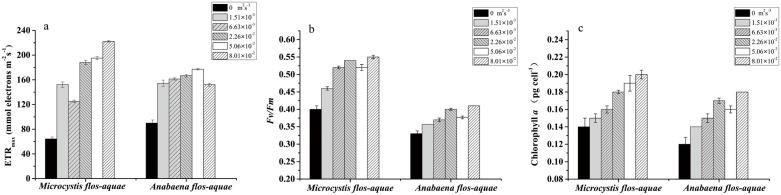
Changes in the maximum electron transfer rate (ETR_max_) (a), *F*_*v*_*/F*_*m*_ (b) and cellular chlorophyll *a* (*Chla*) (c) of *M*. *flos-aquae* and *A*. *flos-aquae* at different turbulent dissipation rate levels in the logarithmic growth phase. The data are the mean ± SD (n = 3).

After incubation with varied levels of turbulent dissipation rates, the maximum electron transport rate (ETR_max_) and the maximum quantum yield (*F*_*v*_*/F*_*m*_) of *M*. *flos-aquae* and *A*. *flos-aquae* showed considerable differences (Fig 2a and 2b). The parameters for the photosynthetic responses to changes in turbulence intensity of *M*. *flos-aquae* were significantly higher than in the control (non-turbulent) treatment (ANOVA, *P* < 0.05). Among all the turbulent mixing treatments, markedly low values of *F*_*v*_*/F*_*m*_ of *M*. *flos-aquae* were found in cells grown at a turbulence intensity of 1.51×10^−3^ m^2^s^-3^. The values of ETR_max_ and *F*_*v*_*/F*_*m*_ were not found to be significantly different among levels of turbulence intensities from 2.26×10^−2^ to 8.01×10^−2^ m^2^s^-3^ (ANOVA, *P* > 0.05). Coincident with the elevation in the ETR_max_ of *M*. *flos-aquae*, the changes in the ETR_max_ of *A*. *flos-aquae* showed similar trends to that of *M*. *flos-aquae* (ANOVA, *P* < 0.05). However, it appears that there was no significant difference in the *F*_*v*_*/F*_*m*_ of *A*. *flos-aquae* between all the turbulent treatments and the controls (ANOVA, *P* > 0.05). The cellular *Chla* showed a similar pattern to the *F*_*v*_/*F*_*m*_ ratios, although the cellular *Chla* was relatively higher at higher turbulent dissipation rates, probably due to the reduction in cell density at the highest turbulent dissipation rates (Fig 2c).

### Phosphate uptake of cyanobacteria

**Fig 3:**
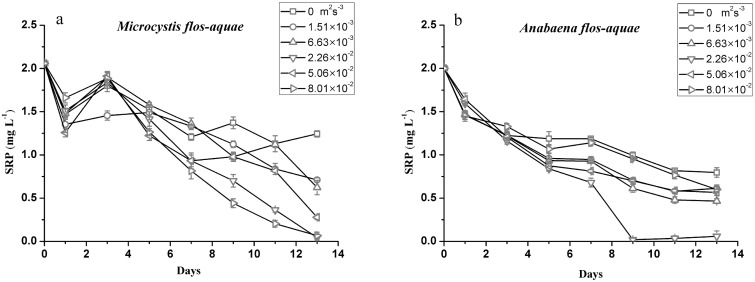
The time course of soluble reactive phosphorus (SRP) in the culture medium of *M*. *flos-aquae* (a) *and A*. *flos-aquae*(b) at different turbulent dissipation rate levels. The data are the mean ± SD (n = 3).

**Fig 4:**
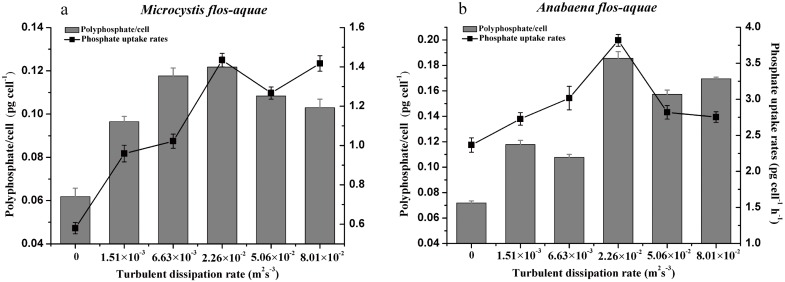
Uptake rate for phosphate and polyphosphate content of *M*. *flos-aquae* (a) *and A*. *flos-aquae* (b) cultured at different turbulent dissipation rate levels in the logarithmic growth phase. The data are the mean ± SD (n = 3).

The time course of SRP in the culture medium and phosphate uptake rate of *M*. *flos-aquae* and *A*. *flos-aquae* cultured at different turbulent dissipation rate levels are shown in Fig 3, respectively. It was observed that the SRP concentrations of the two strains decreased significantly with culturing time at all turbulence intensity levels. The residual P concentrations in the *M*. *flos-aquae* cultures were affected by the turbulent dissipation rate. The concentration of SRP in the cultures treated with higher turbulent dissipation rates (8.01×10^−2^ m^2^s^-3^) were significantly lower than that in the turbulent dissipation rates of 1.51×10^−3^ and 6.63×10^−3^ m^2^s^-3^ at the end of the test (ANOVA, *P* < 0.05). However, the SRP concentration in the *A*. *flos-aquae* culture with a turbulence intensity of 2.26×10^−2^ m^2^s^-3^ was the greatest at the end of the test, whereas no significant differences were observed among the other treatment cultures (ANOVA, *P* > 0.05). The phosphate uptake rate was analyzed as a function of the external phosphate concentration. It showed similar trends to that of SRP (Fig 4). Under the turbulent dissipation rate of 2.26×10^−2^ m^2^s^-3^, the uptake rates of phosphate in the two strains increased with the elevation of turbulence intensity. When the turbulent dissipation rate was above 2.26×10^−2^ m^2^s^-3^, the phosphate uptake rate of *M*. *flos-aquae* remained relatively unchanged, while the phosphate uptake rate of *A*. *flos-aquae* decreased dramatically (ANOVA, *P* < 0.05).

### Effect of turbulence on the elemental composition of cyanobacteria

To further explore the cellular stoichiometry changes, cellular C, N and P in response to different turbulent dissipation rates were analyzed (Table 3). There were significant differences in cellular C, N, and P among different levels of turbulent mixing (ANOVA, P < 0.05). For *M*. *flos-aquae*, cellular C was the highest under the turbulent dissipation rate of 5.06×10^−2^ m^2^s^-3^, with an increase of 11% compared with the control treatment. However, the cellular N concentration was significantly decreased with increasing turbulence intensity, except under the turbulent dissipation rate of 1.51×10^−3^ m^2^s^-3^. Furthermore, as dissipation rates increased, there was also an increase in cellular P, and the maximum value was observed under the turbulent dissipation rate of 2.26×10^−2^ m^2^s^-3^, which was 2.0-fold compared with that at the stagnation condition. Additionally, the changes in cellular PolyP showed similar trends to that of cellular P (Fig 4). In *A*. *flos-aquae*, the highest concentrations of cellular C, N and P were found at the turbulent dissipation of 2.26×10^−2^ m^2^s^-3^. The cellular PolyP kept increasing along with increases in turbulence intensity at the rate of 0–2.26×10^−2^ m^2^s^-3^. Then, a decline of cellular PolyP was observed under the dissipation rate of 5.06×10^−2^ m^2^s^-3^ and above (ANOVA, P < 0.05).

**Table 3:** Cellular stoichiometry of *M*. *flos-aquae* and *A*. *flos-aquae* under different levels of turbulence in the logarithmic growth phase.

Turbulent dissipation rate (m^2^s^-3^)	*M*. *flos-aquae*					*A*. *flos-aquae*				
	pg/cell			mole ratio		pg/cell			mole ratio	
	C	N	P	C/N	N/P	C	N	P	C/N	N/P
**0**	6.14	1.58	0.29	4.53	12.06	6.13	1.38	0.25	5.18	12.22
**1.51×10**^**−3**^	6.70	1.89	0.31	4.13	13.50	7.27	1.48	0.19	5.73	17.24
**6.63×10**^**−3**^	6.73	1.38	0.26	5.68	11.75	6.38	1.31	0.28	5.68	10.35
**2.26×10**^**−2**^	6.36	1.49	0.59	4.97	5.59	8.17	1.55	0.33	6.14	10.40
**5.06×10**^**−2**^	6.89	1.16	0.43	6.92	5.97	6.81	1.38	0.21	5.75	14.55
**8.01×10**^**−2**^	5.52	0.91	0.33	7.07	6.11	7.22	1.49	0.2	5.65	16.49

Changes in molar ratios of C/N and N/P of *M*. *flos-aquae* and *A*. *flos-aquae* are also shown in Table 3. The results from our experiment showed plasticity of cellular ratios in *M*. *flos-aquae* and *A*. *flos-aquae* in response to small-scale turbulence. There was an increase in the C/N ratio and a decrease in the N/P ratio in *M*. *flos-aquae* at the dissipation rate of 6.63×10^−3^ m^2^s^-3^ and above, compared with the controls. Variations in the C/N ratio in *A*. *flos-aquae* were similar to that in *M*. *flos-aquae*. The maximum C/N ratio was recorded at the turbulent dissipation rate of 2.26×10^−2^ m^2^/s^3^. However, the N/P ratio in *A*. *flos-aquae* decreased only at the turbulent dissipation rates of 6.63×10^−3^ and 2.26×10^−2^ m^2^s^-3^, compared with the control treatment, which was due to the increase in cellular P.

### The filament length of *A*. *flos-aquae*

**Fig 5:**
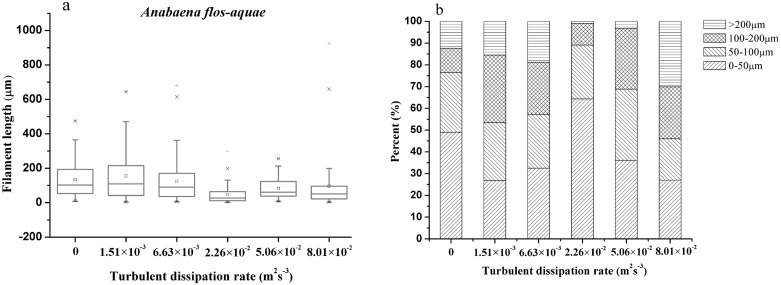
Box and whisker plots (a) and frequency distribution (b) of *A*. *flos-aquae* filament length as a response to various turbulence intensities at the end of the test. a, filament lengths are presented in box plots showing the 5, 10, 25, 50, 75, 90 and 95 percentiles; whiskers represent minimum and maximum values, and dots inside the boxes are the mean values.

The filament length of *A*. *flos-aquae* varied with different turbulent dissipation rates (Fig 5a). It was observed that the geometric mean of the filament lengths of *A*. *flos-aquae* decreased with the elevation of turbulence intensity. Compared with the stagnation condition, the filament length of *A*. *flos-aquae* decreased by 69% and 47% at the turbulent dissipation rates of 2.26×10^−2^ and 5.06×10^−2^ m^2^s^-3^, respectively. The frequency distribution of each filament length range under the different turbulence intensities is shown in Fig 5b. The result also demonstrated that the frequency of 0–50 μm lengths was increased with increasing dissipation rates and reached the maximum at the 2.26×10^−2^ m^2^s^-3^, while the large filament (>200 μm) was the lowest. The frequencies of 100–200 μm lengths decreased at the turbulent dissipation rates of 1.51×10^−3^ and 6.63×10^−3^ m^2^s^-3^, while for the high turbulence intensities of 8.01×10^−2^ m^2^s^-3^, the frequencies of large filaments (>200 μm) also decreased significantly.

## Discussion

Turbulence is ubiquitous in aquatic systems and thus can potentially influence a wide scope of phytoplankton and eco-physiological processes. It is still often referred to as one of the unsolved problems in field studies due to the interactions with other variables, such as light penetration and supply of nutrients, and their consequences for organism physiology and behaviors [44]. Some of the current knowledge has been derived from laboratories with devices to generate controlled turbulence; however, these should correctly evaluate the response of phytoplankton to a certain level of turbulence in natural conditions [45]. A gradient of increasing levels of dissipation rates was used in our study to investigate the shape and character of potential relationships between small-scale turbulence, nutrient conditions and cyanobacteria. Turbulent dissipation in the device varied from 10^−4^ to 10^−2^ m^2^s^-3^. On the Kolmogorov microscale, it was in the higher range of turbulences in natural aquatic ecosystems. However, it covered the range of turbulence intensities experienced by cyanobacteria in the field [2].

In most studies described in the literature to date, the effect of turbulence on phytoplankton was determined by changes in growth and the viability of the organism [46, 47]. In this study, responses of *Microcystis* and *Anabaena* to turbulence intensities were quite different as inferred by growth. The turbulent regimes of 1.51×10^−3^ to 2.26×10^−2^ m^2^s^-3^ favored the growth of the two strains. However, in regard to the higher dissipation rate of 8.01×10^−2^ m^2^s^-3^, turbulence had a negative impact on the growth rate of *A*. *flos-aquae* but did not affect the growth of *M*. *flos-aquae*, suggesting that unicellular *M*. *flos-aquae* was better adapted to growing under high turbulent dissipation rates.

The photosynthetic system is a good indicator system because it is very sensitive to any changes in living conditions [48]. *F*_*v*_*/F*_*m*_ and ETR_max_ are considered as the sensitive photosynthetic parameters to environmental stress [49]. At the used range of turbulent mixing, the changes in *F*_*v*_*/F*_*m*_ and ETR_max_ in the exponential phase support the hypothesis that the photosynthetic activity of *A*. *flos-aquae* significantly increases when compared with stagnant conditions but might not change in contrast to the unimodal response of a growth rate to a gradient of turbulent mixing. Similar results were also discussed by [50], indicating that at saturating irradiances, photosynthesis was less sensitive than growth to changes in turbulence mixing. Additionally, the *Chla* content in both *M*. *flos-aquae* and *A*. *flos-aquae* increased with turbulence even under a higher level, indicating that the photosynthetic apparatus of algae showed little or no disruption in response to turbulence. Therefore, the potential indirect effects of a light regime under the gradient of turbulent mixing, e.g., the shading effect due to an increase in the population in the reactors, could be ignored.

A great majority of studies reported that turbulence may have beneficial effects on phytoplankton due to an increase in the diffusive transport of nutrients toward the cell surface and, hence, an increase the nutrient uptake rate [14, 51]. Under turbulent mixing in our study, cellular N and C of the two stains showed approximately the same responses, resulting in a similar pattern of C/N ratios in response to turbulent mixing. Unlike cellular C and N, P uptake and assimilation in cells did not show a unimodal response. Turbulence is reported to affect the algal growth by changing their nutrient acquisition traits [52]. The fact that the change in phosphate uptake rates was similar to that of growth in both of the strains implied that different growth characteristic responses to turbulence may be dependent on the P strategy. This finding was consistent with previous research [47] in that the phosphate uptake rate of algae was approximately similar to the growth rate.

Formation and storage of PolyP is commonly regarded as a nutritional strategy of microalgae when encountering environmental stress [53]. According to Fig 4, it was shown that luxury uptake of phosphorus by cyanobacteria was mostly applied to accumulate PolyP, implying that accumulation of PolyP at high dissipation rates was the strategy for cyanobacteria to maintain phosphorus concentrations in unfavorable conditions. However, compared with *M*. *flos-aquae*, the concentration of PolyP in *A*. *flos-aquae* at dissipation rates of 2.26×10^−2^ to 5.06×10^−2^ m^2^s^-3^ were much higher than those with a dissipation rate of 1.51×10^−3^ m^2^s^-3^, indicating that the reallocation of cellular P in *A*. *flos-aquae* could be a response to the changed turbulent conditions, and the filament integrity in *A*. *flos-aquae* was more sensitive to turbulence than in unicellular *M*. *flos-aquae*. Furthermore, our results also showed that the filament length of *A*. *flos-aquae* decreased in response to turbulence. It seemed that turbulence negatively affects cyanobacterial morphology prior to physiological activity. We stress that this may be related to the nutrient acquisition strategies, which was depended on algal size. Theoretical predictions regarding the thickness of external diffusion boundary layer is equal to the equivalent spherical diameters of the organism [54]. Thus, larger organism increased the restriction on nutrient uptake from low external concentrations, probably because the greater thickness of the diffusion boundary layers around itself. As small filaments have higher rates of nutrient uptake and lower metabolic requirement, which allow them to grow at much lower resource concentrations than larger ones [55], we inferred that with the exhaustion of SRP in the culture, the decreased filaments of *A*. *flos-aquae* at higher turbulent dissipation rates could favor the resource gain. Our findings support what had been suggested by [47], such that, in the lotic diatoms where the cells were smaller, there was higher P affinity. Overall, our study also highlights the need for further investigation of the relationship between the boundary layer around filaments and turbulence mixing.

A gradient of increasing levels of dissipation rates is useful when attempting to investigate the shape and character of potential relationships between small-scale turbulence, nutrient conditions and planktonic organisms [56]. Small-scale shearing affects physiological activities and the morphology of phytoplankton in high turbulence environments. The variety of physiological and morphological properties of phytoplankton may be largely considered as adaptations to different scales and characteristics of turbulent motions in the aquatic environment [57]. In concert with earlier investigations, the results from the two types of cyanobacteria in our study illustrated that both *M*. *flos-aquae* and *A*. *flos-aquae* adjust their growth rates in response to turbulence levels in the ways of asynchronous cellular stoichiometry of C, N, and P, especially the phosphorus strategy, to improve the nutrient application efficiency. Additionally, the colonial *A*. *flos-aquae* could change the effective filament length to enhance the nutrient uptake under high turbulent conditions. Again, these evidences supported the hypothesis that hydrodynamic regimes may impact eco-physiology of cyanobacteria to form certain adaptive strategies, which should be carefully considered in elucidating mechanisms of cyanobacteria blooms. Clearly, future studies are warranted to assess the effects of turbulence on nutrient flux of cells including different morphological cyanobacteria species.

## Supporting Information

S1 File The validation results of CFD modeling and PIV measurement of flow pattern. (DOCX)
