# Supplementary material for: Effect of Small-Scale Turbulence on the Physiology and Morphology of Two Bloom-Forming Cyanobacteria
Source: PLoS One. 2016 Dec 30;11(12):e0168925. doi: 10.1371/journal.pone.0168925 (PMC5201306; doi:10.1371/journal.pone.0168925)
Supplement: S1 File — (DOCX) [file pone.0168925.s001.docx]

**Supporting information**

**Result of model validation**

Model validation was conducted by comparing the axial velocities at r=0.033m and radial velocities at z=0.054m obtained from simulation with those from the experiments. Figure S1a-d shows that the axial and radial velocities obtained from CFD match well with the PIV data, and that both predictions and experiments obey the flow profile formed by one radial impeller. As shown in Table S1, the simulated turbulent dissipation rate is about 10% lower than the measured value, which is generally acceptable since the turbulence models[^1^](#_ENREF_3) and the energy dissipation rate calculation methodologies[^2^](#_ENREF_4) will affect the simulated and measured turbulent parameters respectively. All the data indicates the simulation results are reliable.

For a specific impeller, the power consumption (P, W) correlation in terms of the impeller diameter (D, m) and rotation speed (N, r/s) is given as

*P=N_P_ρN^3^D^5^*  (1)

Where *N_p_* is power number and *ρ* is medium density. Figure S2 shows the simulated power consumptions of the impeller in different rotation speeds and fitting curve by equation 1. It can be seen that the equation fits well with the simulated values, and the correlation coefficient is 0.993, which also indicates the reliability of the simulations to a certain extent.


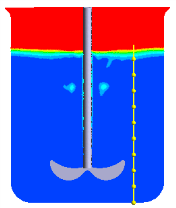

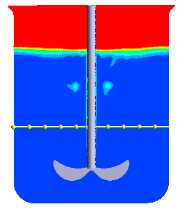

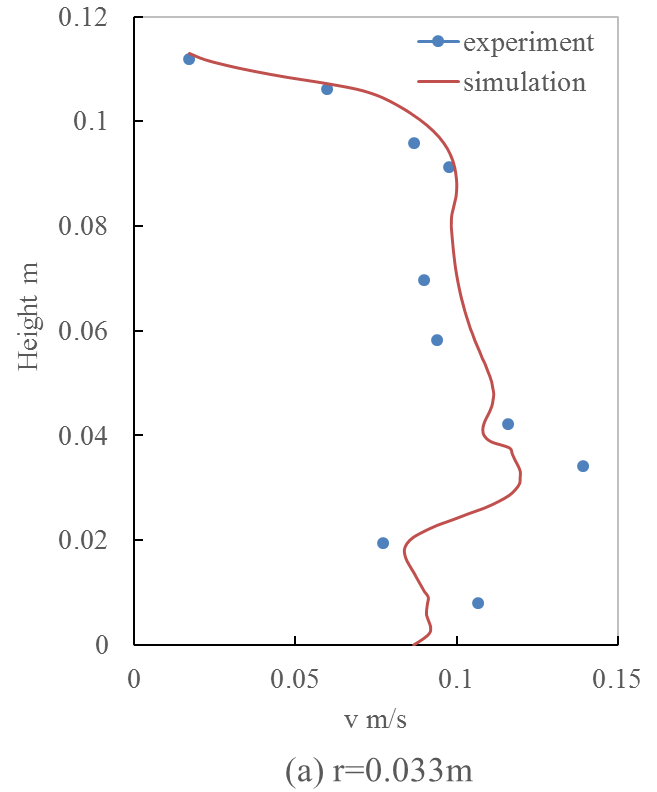

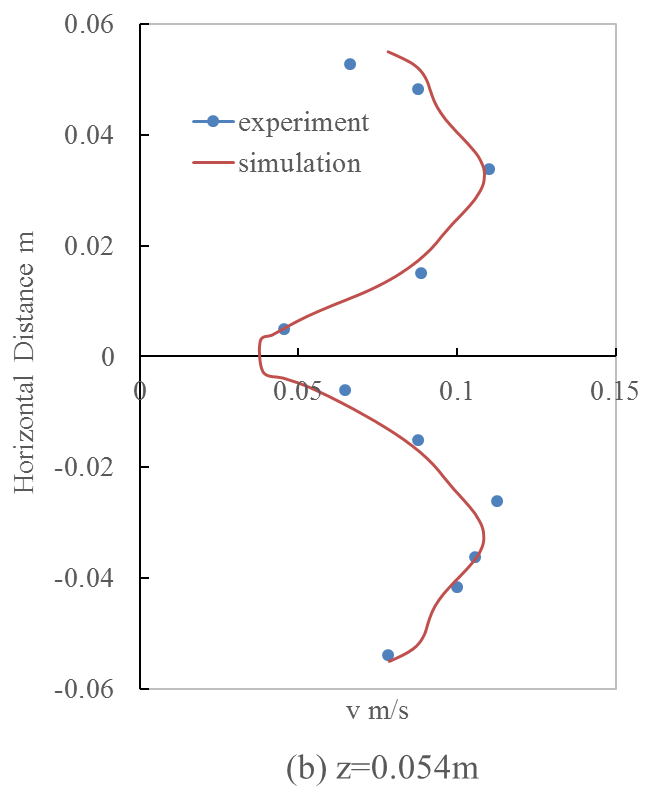


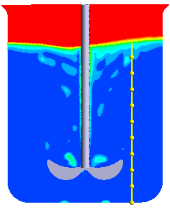

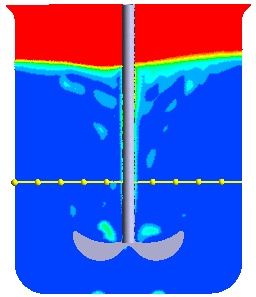

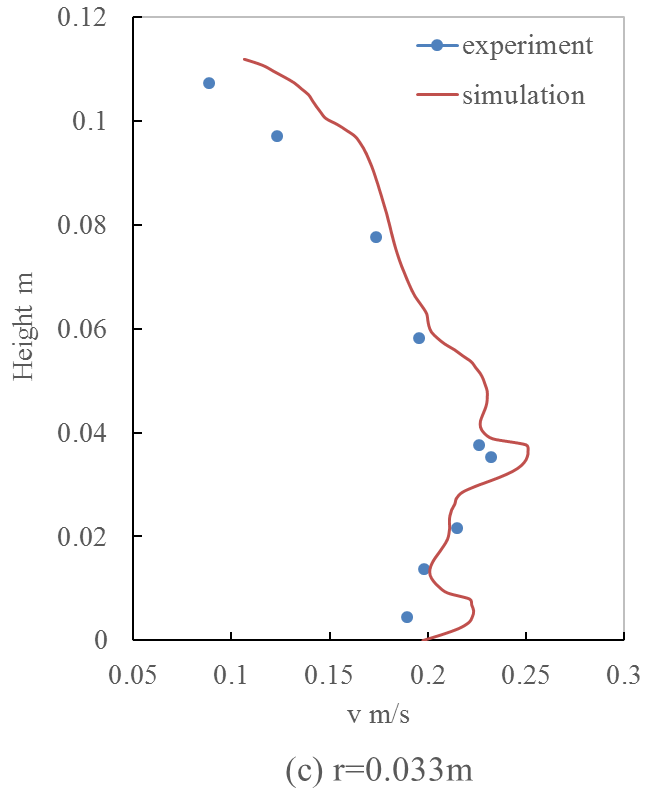

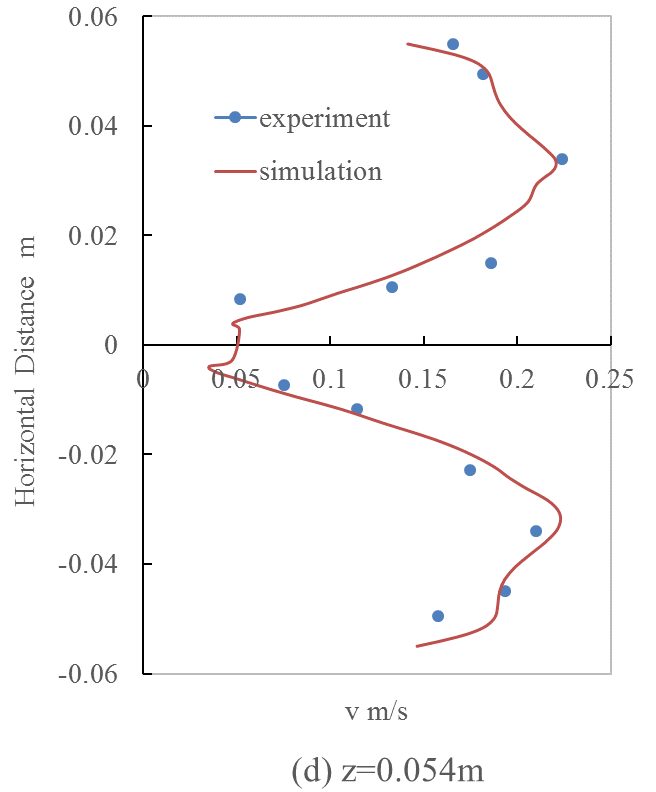


Figure S1. Comparison of velocities obtained from experiment and CFD simulation at axial and radial position (a: 100rpm, axial; b: 100rpm, radial; c: 200rpm, axial; d: 200rpm, radial)


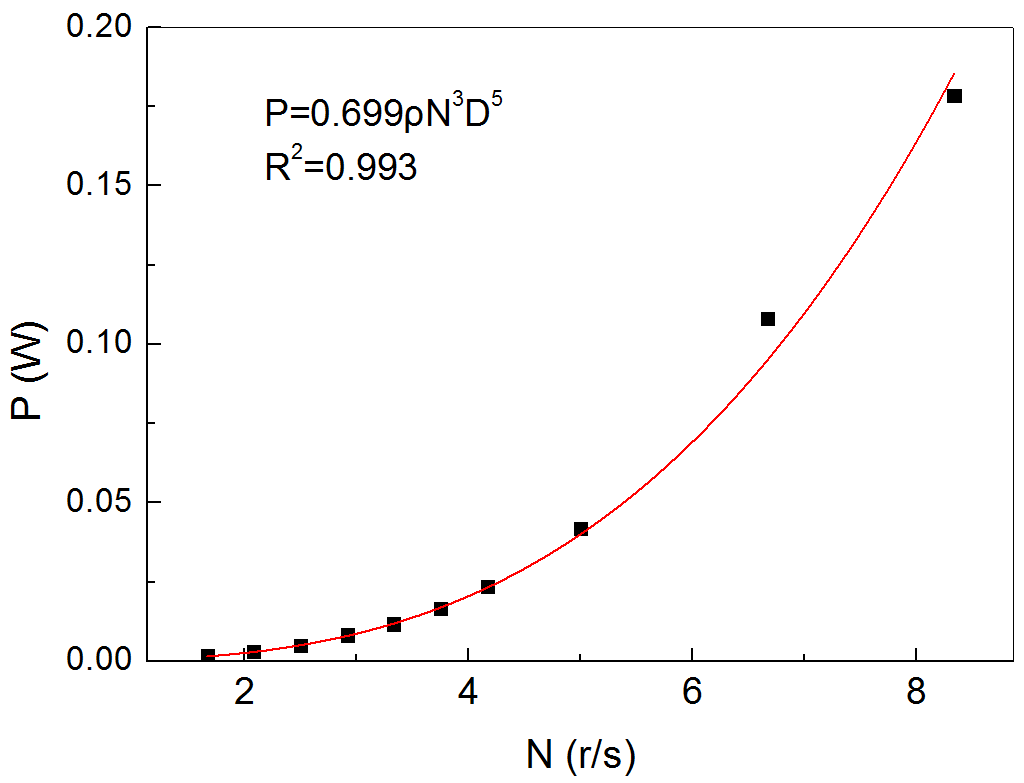


Figure S2. The simulated power consumptions of the impeller in different rotation speeds.

Table S1. Comparison of volume-averaged turbulent dissipation rates obtained from experiment and CFD simulation

|  | PIV Experiment | | Simulation | | | | |
| --- | --- | --- | --- | --- | --- | --- | --- |
|  | 100 rpm | 200 rpm | 100 rpm | 200 rpm | 300rpm | 400rpm | 500rpm |
| Turbulent dissipation rates (m^2^/s^3^) | 0.00172 | 0.00714 | 0.00151 | 0.00663 | 0.02259 | 0.05058 | 0.08010 |

1. B. Wu, *Biotechnol. Bioeng.*, 2012, **109**, 804-812.

2. M. M. Hoque, M. J. Sathe, S. Mitra, J. B. Joshi and G. M. Evans, *ChEnS*, 2015, **137**, 752-767.
